# Continuing bonds in men grieving a loved one

**DOI:** 10.1590/1518-8345.6753.4011

**Published:** 2023-10-06

**Authors:** Daniel Martínez-Esquivel, Derby Muñoz-Rojas, Alfonso Miguel García-Hernández

**Affiliations:** 1 Universidad de La Laguna, San Cristóbal de La Laguna, Santa Cruz de Tenerife, España.; 2 Becario de la Universidad de Costa Rica, Costa Rica.; 3 Universidad de Costa Rica, Escuela de Enfermería, Montes de Oca, San José, Costa Rica.

**Keywords:** Psychological Adaptation, Cause of Death, Men, Grief, Death, Bereavement, Adaptación Psicológica, Causas de Muerte, Hombres, Pesar, Muerte, Aflicción, Adaptação Psicológica, Causas de Morte, Homens, Luto, Morte, Pesar

## Abstract

**Objective:**

to examine internalized and externalized continuing bonds in men grieving a loved one.

**Method:**

a correlational, descriptive and cross-sectional study. Convenience sample comprised by 170 mourning men. The variables were mediators of mourning, continuing bonds and sociodemographic data. The instrument used was an online questionnaire comprised by mediators of mourning, a continuing bonds scale and sociodemographic data. Descriptive statistics, analysis of variance and Spearman’s coefficient were used. The significance level adopted was p<0.05.

**Results:**

the participants’ mean age was 36.61 years old (SD=13.40), and 80.00% had Higher Education. The mean values corresponding to internalized and externalized continuing bonds were 24.85 (SD=7.93) and 7.68 (SD=2.33), respectively. Significant differences were established referring to internalized and externalized continuing bonds in terms of kinship with the deceased person (p<0.001), and none with the cause of death or with the time elapsed since the event. No significant correlations were defined between internalized/externalized continuing bonds and mediators of mourning.

**Conclusion:**

grieving men express internalized and externalized continuing bonds frequently and occasionally, respectively, with differences according to who the deceased person was. The Nursing discipline might devise specific strategies that strengthen coping with grief in this population group.

## Abstract (es)

**Objetivo:**

examinar la continuidad de vínculos interna y externa en hombres que experiencian duelo por un ser querido.

**Método:**

estudio correlacional, descriptivo y transversal. Muestra a conveniencia de 170 hombres dolientes. Las variables fueron mediadores del duelo, continuidad de vínculos y datos sociodemográficos. Se utilizó un cuestionario en línea compuesto por mediadores de duelo, escala de continuidad de vínculos y datos sociodemográficos. Se empleó estadística descriptiva, análisis de varianza y coeficiente de Spearman. El nivel de significancia correspondió a p<0,05.

**Resultados:**

la media de edad de los participantes fue de 36,61 años (DE=13,40), y el 80,00% tenía educación superior. Los valores medios de continuidad de vínculos interna y externa fueron 24,85 (DE=7,93) y 7,68 (DE=2,33), respectivamente. Se establecieron diferencias significativas referentes a la continuidad de vínculos interna y externa entre parentesco de la persona fallecida (p<0,001), y ninguna con la causa de muerte o con el tiempo transcurrido desde el fallecimiento. No se precisaron correlaciones significativas entre continuidad de vínculos interna/externa y mediadores del duelo.

**Conclusión:**

los hombres dolientes expresan la continuidad de vínculos interna de manera frecuente y la externa en ocasiones, con diferencias respecto a quién era la persona fallecida. La Enfermería podría diseñar estrategias específicas que fortalezcan el afrontamiento del duelo en este grupo.

## Abstract (pt)

**Objetivo:**

examinar a manutenção de vínculos interna e externa em homens vivenciando o luto por um ser querido.

**Método:**

estudo correlacional, descritivo e de corte transversal. Amostra de conveniência de 170 homens em luto. As variáveis foram: mediadores do luto, manutenção de vínculos e dados sociodemográficos. Utilizou-se um questionário *online* composto por mediadores de luto, escala de manutenção de vínculos e dados sociodemográficos. Empregou-se estatística descritiva, análise de variância e coeficiente de Spearman. Nível de significância p<0,05.

**Resultados:**

os participantes tinham uma média de idade de 36,61 anos (DP=13,40) e 80,00% tinham ensino superior. A média de manutenção interna dos vínculos foi de 24,85 (DP=7,93) e a de manutenção externa foi de 7,68 (DP=2,33). Foram estabelecidas diferenças significativas para a manutenção dos vínculos internos e externos entre os parentes do falecido (p<0,001), nenhuma com a causa da morte ou o tempo decorrido desde a morte. Não foram encontradas correlações significativas entre a manutenção dos vínculos internos e externos e os mediadores do luto.

**Conclusão:**

os homens em luto expressaram a manutenção interna dos vínculos com frequência e a manutenção externa dos vínculos ocasionalmente, com diferenças a respeito de quem era a pessoa falecida. A enfermagem poderia criar estratégias específicas para fortalecer o enfrentamento do luto nesse grupo.

**Highlights:**

**(1)** It was usual to continue the bond with the deceased loved one.

**(2)** The expression of internalized continuing bonds was frequent after the death.

**(3)** When facing the loss, there was occasional expression of externalized continuing bonds.

**(4)** There were differences in internalized and externalized continuing bonds according to kinship.

**(5)** There were no associations between continuing bonds and mediators of mourning.

## Introduction

The death of a loved one is a profoundly challenging event for each person, where personal, interpersonal and environmental resources are put to the test. This challenge is mediated by grief as a single and individual answer characterized by elements with distinctive sociocultural meanings. Consequently, two or more people might not experience the same in relation to their thoughts, behaviors or feelings^(^ 1 ^)^.

Considering men, they have been singularized under the social construct of masculinity that dictates a series of beliefs and expectations about how they should behave in society. Traditional masculinity is an ideology which asserts that men are governed by norms, perhaps stereotyped, and defined as dominant beings, physically strong, violent and homophobic, among other attributes, typecasting them into a social status marked by precariousness^(^ 2 ^)^.

However, certain transformation has been perceived that includes changes in the masculinity traits where men assume roles related to self-care and to caring for others. In addition, qualities such as empathy, communication, affection and cooperation are gaining social value for this collective. As a consequence, different types of men would be configured according to how their identity is elaborated, which might exert an influence on the way in which they face grief^(^ 3 ^)^.

Given the above, there is wide range of masculinities, reason why this study considered all people that self-identify as men. However, when observing a phenomenon such as grief, no discrimination based on sex or gender should exist but according to each person’s coping resources, so that they ease (or hinder) the adjustment and adaptation process facing the loss^(^ 4 ^)^.

Therefore, grief is an adaptation process to a new reality that emerges as the result of the loss, actual or perceived, of a loved one. It is a natural and self-limited human response with a series of physical, cognitive and behavioral manifestations or with injuries affecting the grieving person’s health. It is an intersubjective, specific and intense experience determined by several factors known as mediators of mourning^(^ 5 ^)^.

Mediators of mourning ease interpreting the reason why people follow different paths in the grief experience according to their personal stories and meanings. They describe the deceased loved one’s characteristics and what it is that affects the grieving person. They are divided as follows: who was the deceased person?; nature of the bond; how the person died, historical background; personality variables; social variables; and concurrent tensions. Each mediator has a series of components; however, kinship with the deceased person, cause of death and time elapsed since the event were included in this research^(^ 6 ^)^.

Needless to say, Continuing Bonds (CBs) represent a normal aspect of the grief process that emphasizes the continuous relationship with the deceased loved one in the face of their physical absence. They would manifest as a persistent commitment to the memories and images of the deceased person; they are not mere ideas or feelings. They would generate consolation, although they depend on their essence for that. It is considered that CBs are not linear, as they present different expression modalities within which the internalized and externalized ones have been distinguished^(^ 7 ^)^.

Internalized CBs correspond to mental representations that turn the deceased person into safe grounds that ease acknowledging death. Externalized CBs include erroneous perceptions about the reality of death, causing rejection that would give rise to a negative conflict. The contribution of internalized and externalized CBs to the grief process is hard to determine; however, the social, cultural, political and religious heritage needs to be considered to contextualize such connection^(^ 8 ^)^.

Previous literature has already studied the grief process as CBs, both internalized and externalized, with diverse populations and in various contexts. In order to illustrate this, after analyzing CBs in men and women who lost a loved one, it was pointed out that internalized CBs are a mediator of kinship with the deceased person and that externalized CBs mediate the cause of death, both with grief adjustment as a result^(^ 9 ^)^.

In the case of parents who lost a child, internalized CBs had significant and significant and negative correlations with cause of death; in opposition, externalized CBs evidenced positive correlations with this grief mediator^(^ 10 ^)^. In adults, it might be predicted if CBs are adaptive or non-adaptive. Furthermore, regardless of whether CBs are internalized or externalized, the time elapsed since the event would increase the grief symptoms^(^ 11 ^)^.

Previous results exemplify the relevance of associating CBs with other variables to better understand them so that they guide new care proposals for the population. In the understanding that has highlighted the major contribution of women, it has been argued that the traditional theories about grief are grounded on female coping styles. Various research papers have recommended developing surveys with greater male participation or that only include men^(^ 11 ^-^ 12 ^)^.

Added to the fact that the grief process as CBs has not been recently investigated specifically in men, the aforementioned validates development of this project, which seeks to understand men’s experiences regarding this topic. In view of this, the objective proposed is to examine internalized and externalized continuing bonds in men grieving a loved one. On the other hand, it is sought to answer the following hypotheses:

H_1_: There are differences between mediators of mourning over internalized and externalized continuing bonds among men grieving a loved one.
H_2_: There are relationships between internalized and externalized continuing bonds and mediators of mourning among men grieving a loved one.

## Method

The text followed the guidelines for the communication of observational studies set forth in the Strengthening the Reporting of Observational studies in Epidemiology (STROBE) guide^(^ 13 ^)^.

### Design

A non-experimental, correlational, descriptive and cross-sectional study was conducted about grief as CBs in men^(^ 14 ^)^.

### Locus

The study was developed in Costa Rica, in the online modality. Costa Rica is a Central American country. Its territory is 51,100 km^2^ in extension and is divided into 7 provinces, with San José standing out as its capital city. The estimated total population is 5,213,362 inhabitants and, in particular, 2,624,989 would correspond to men^(^ 15 ^)^.

### Period

Data collection was carried out during 2022.

### Population

The study population was comprised by all grieving men in Costa Rica.

### Selection criteria

The following inclusion criteria were considered: identifying as a man; being at least 18 years old; being a Costa Rican; living in Costa Rica; and having suffered the loss of a loved one, regardless of the time elapsed. Regarding the exclusion criterion, it was delimited to undergoing Mental Health Nursing, Psychology or Psychiatry therapy for the treatment of grief. It is noted that no information from those who failed to answer the entire data collection instrument was included.

### Definition of the sample

The sample was for convenience, which ensured careful selection of the participants guided by the characteristics of the research study. With an *a priori* analysis in the G.Power 3.1.9.2 software with size effect of 0.5, alpha of 0.05 and power of 0.95, sample size was calculated at 115 and was overestimated by 30%, which represented another 34 individuals, defining a sample comprised by 149 subjects. Finally, 170 grieving men (n=170) took part in this research.

### Study variables

Variables such as the following were chosen: mediators of mourning (kinship with the deceased person, cause of death, time elapsed since the event); CBs (internalized and externalized); and sociodemographic data (age, place of residence, schooling level, employment situation).

### Instruments used to collect the information

The data were collected using a self-applied online questionnaire that was prepared on the LimeSurvey platform, a professional tool for online data collection. It was divided in three aspects:

1. Mediators of mourning, which identified the degree of kinship with the deceased person, the cause of death and the time elapsed since the event.
2. *Escala de Continuidad de Vínculos* (ECoVin), translated from the Continuing Bonds Scale^(^ 9 ^)^. It assessed the bond with a loved one after their death. The scale was adapted and validated for Spanish with an excellent internal consistency level (*α*=0.914)^(^ 16 ^)^. It includes 16 items such as the following: “I have thought about the deceased person as a role model” or “Even if for a moment, I have come to mistake other people with the deceased”, which resort to a Likert scale with 4 possible answers scored from 1 to 4, where 1 is “Not at all” and 4 is “Constantly”. Higher scores indicate greater bonds with the deceased loved one; the minimum is 16 and the maximum, 64. It has two subscales that assess internalized CBs (10 items) with scores from 10 to 40, and externalized CBs (6 items) with values from 6 to 24. In this study, a Cronbach’s Alpha of 0.898 was reported for the entire scale, as well as *α*=0.916 and *α*=0.717 for the internalized and externalized CBs subscales, respectively.
3. The sociodemographic data distinguished aged, place of residence, schooling level and employment situation.

### Data collection

For the recruitment phase, an invitation was created with a motivating reason to take part in the study, the link to the questionnaire, a QR code with the contact data, and an infographic with basic information about the research. The materials were sent to the people in charge of several institutions or organizations for open diffusion in support groups for men, support groups for grief management, and public interaction in social networks or in other messaging platforms. There was no access to the participants’ contact data and personal information.

### Data treatment and analysis

The data were downloaded and screened to prepare a database in Microsoft Excel 16.69.1, which was exported to the Statistical Package for the Social Sciences 25 (IBM SPSS 25) to run the statistical analysis in charge of the researchers and refine precision. In order to establish the characteristics of the sample, descriptive statistics was used when quantifying frequency distributions, central tendency measures and variability measures. Inferential statistics was used: to determine differences between the groups, the Analysis of Variance (ANOVA) test was calculated and, for the associations between the variables, Spearman’s correlation. The significance level adopted was p<0.05. Reliability of ECoVin was analyzed with Cronbach’s Alpha. No missing values were reported.

### Ethical aspects

Privacy and confidentiality of all the participants was guaranteed. Each individual had access to an informed consent form to agree or refuse to collaborate. Development of the study was based on ethical principles and good research practices to ensure respect for human rights. The protocol was approved by the Scientific Ethics Committee (*Comité* Ético *Científico*) belonging to the University of Costa Rica (CEC-259-2022).

## Results

The sample comprised by grieving men (n=170) had a mean age of 36.61 years old (SD=13.40). In relation to schooling, 80.00% (n=136) had Higher Education, whereas 17.60% (n=30) had High School and 2.40% (n=4), Elementary School. In no case were lower levels reported. Most of the participants (74.10%) reported having a job (n=126), 18.20% were unemployed (n=31) and the others were devoted to other activities (n=13). The rest of the characteristics can be checked in Table 1.

**Table 1 -:** Distribution of the sociodemographic data and mediators of mourning in men grieving a loved one (n=170). Costa Rica, 2022

Variable		Grieving men (***n*=**170)	
		***fi***	% valid
Age	18-30 years old	70	41.20
	31-40 years old	48	28.20
	41-50 years old	19	11.20
	51-60 years old	22	12.90
	61-70 years old	11	6.50
Place of residence	Alajuela	21	12.40
	Cartago	29	17.10
	Guanacaste	2	1.10
	Heredia	28	16.50
	Limón	1	0.60
	Puntarenas	3	1.70
	San José	86	50.60
Kinship with the deceased person	Grandfather/Grandmother	59	34.70
	Father/Mother	57	33.50
	Sibling	11	6.50
	Son/Daughter	14	8.30
	Partner	5	2.90
	Friend	6	3.50
	Other	18	10.60
Cause of death	Cancer	63	37.20
	Chronic disease	52	30.60
	Acute disease	30	17.60
	Accident	13	7.60
	Perinatal death	0	0.00
	Homicide	6	3.50
	Suicide	6	3.50
Time elapsed since the event	3 months or less	16	9.40
	Between 3 and 6 months	8	4.70
	Between 6 and 9 months	5	2.90
	Between 9 months and 1 year	7	4.10
	Between 1 and 2 years	17	10.00
	Between 2 and 5 years	46	27.20
	Between 5 and 10 years	31	18.20
	Between 10 and 20 years	26	15.30
	More than 20 years	14	8.20

The results related to CBs had a mean of 32.52 (SD=9.15), with mean values of 24.85 (SD=7.93) in internalized CBs and 7.68 (SD=2.33) in externalized CBs. After making the comparative investigation by mediators of mourning, significant differences were identified between kinship with the deceased person and internalized and externalized CBs (Table 2).

**Table 2 -:** Comparison according to internalized and externalized continuing bonds by kinship with the deceased person in men grieving a loved one (n=170). Costa Rica, 2022

	Grandfather/ Grandmother (n=59)	Father/ Mother (n=57)	Sibling (n=11)	Son/ Daughter (n=14)	Partner (n=5)	Friend (n=6)	Others (n=18)	p-value
	X* (SD)†	X* (SD)†	X* (SD)†	X* (SD)†	X* (SD)†	X* (SD)†	X* (SD)†	
Internalized CBs‡	23.29 (7.84)	27.98 (7.11)	19.00 (6.79)	27.93 (6.15)	29.00 (8.21)	25.00 (8.80)	20.00 (7.23)	0.001
Externalized CBs‡	7.39 (1.80)	7.74 (2.44)	7.09 (1.81)	7.57 (1.95)	11.80 (4.08)	10.67 (3.88)	6.72 (0.75)	0.001

*X = Mean; †SD = Standard Deviation; ‡CBs = Continuing Bonds

Regarding the other mediators of mourning, no significant differences were found referring to cause of death (Table 3) or time elapsed since the event (Table 4), both in internalized and externalized CBs.

**Table 3 -:** Comparison according to internalized and externalized continuing bonds by cause of death in men grieving a loved one (n=170). Costa Rica, 2022

	Cancer (n=63)	Chronic disease (n=52)	Acute disease (n=30)	Accident (n=13)	Homicide (n=6)	Suicide (n=6)	p-value
	X* (SD)†	X* (SD)†	X* (SD)†	X* (SD)†	X* (SD)†	X* (SD)†	
Internalized CBs‡	24.84 (9.02)	25.13 (6.42)	26.07 (7.36)	22.69 (9.89)	24.00 (7.12)	21.83 (7.93)	0.756
Externalized CBs‡	7.81 (2.60)	7.33 (1.76)	7.50 (2.22)	8.31 (2.72)	9.17 (3.86)	8.33 (1.86)	0.670

*X = Mean; †SD = Standard Deviation; ‡CBs = Continuing Bonds

**Table 4 -:** Comparison according to internalized and externalized continuing bonds by time elapsed since the event in men grieving a loved one (n=170). Costa Rica, 2022

	3 months or less (n=16)	Between 3 and 6 months (n=8)	Between 6 and 9 months (n=5)	Between 9 months and 1 year (n=7)	Between 1 and 2 years (n=17)	Between 2 and 5 years (n=46)	Between 5 and 10 years (n=31)	Between 10 and 20 years (n=26)	More than 20 years (n=14)	p-value
	X* (SD)†	X* (SD)†	X* (SD)†	X* (SD)†	X* (SD)†	X* (SD)†	X* (SD)†	X* (SD)†	X* (SD)†	
Internalized CBs‡	22.50 (8.51)	29.25 (8.27)	25.20 (6.61)	26.29 (8.78)	25.59 (7.15)	25.07 (7.55)	23.87 (7.65)	24.31 (8.20)	25.71 (9.92)	0.761
Externalized CBs‡	8.25 (3.35)	8.25 (2.55)	6.80 (0.83)	7.14 (2.26)	8.24 (2.41)	7.63 (1.75)	7.48 (1.45)	7.23 (2.40)	8.00 (3.98)	0.772

*X = Mean; †SD = Standard Deviation; ‡CBs = Continuing Bonds

No significant relationships were detected when examining the associations between internalized/externalized CBs and mediators of mourning. In an additional analysis between CBs and mediators of mourning, no significant correlations were either detected (Table 5).

**Table 5 -:** Correlation between continuing bonds and mediators of mourning in men grieving a loved one (n=170). Costa Rica, 2022

Variable	CBs*	Kinship with the deceased person	Cause of death	Time elapsed since the event
CBs*	1			
Kinship with the deceased person	0.027	1		
Cause of death	-0.006	0.075	1	
Time elapsed since the event	-0.063	-0.157‡	-0.079	1

*CBs = Continuing Bonds; †p<0.01 (two-tailed); ‡p<0.05 (two-tailed)

## Discussion

The results about CBs in men grieving a loved one might provide evidence to meet the objective and hypotheses set forth. It might be stated that the internal bonds with the deceased person are expressed frequently and the external ones, occasionally. In addition, there differences regarding internalized and externalized CBs when they are compared in terms of kinship with the deceased person, which is not the case in the comparison to cause of death and time elapsed since the event. Likewise, it can be stated that there are no significant correlations between CBs and mediators of mourning. Therefore, H_1_ would be partially accepted whereas H_2_ would be rejected.

In relation to grief management in men, it has been qualified by the use of intellectualization as a defense mechanism for their affects, by guilt towards the inability to protect the dead person, by anger for what happened or by devoting time to activities. Without necessarily defining if they are suitable standards, it would seem that men focus their loss experiences on loneliness and in problem-solving. However, men’s attitudes towards grief should not be spared on without considering the context and circumstances around the event, especially when most grieving people incorporate the loss to the new reality without the loved one in a brief period of time with no complications^(^ 17 ^)^.

These appraisals are opposed to the value of emotional expressions and to the insistent search for emotional support to cope with grief, which has been traditionally associated with female roles. Despite that, it is deemed opportune to avoid strict delimitations of masculinity and to focus on the effect of the different coping styles when facing distress. A possible reflection is that there is no single path in grief adjustment, with the results representing a reflex of the plurality of the participants’ biographies and masculinities^(^ 18 ^)^.

For the grieving men who took part in the research, the magnitude with which CBs are expressed would reveal the importance of remaining habitually linked to deceased loved ones. Internalized CBs were frequently expressed and externalized ones, occasionally. This is in line with other results which have interpreted that connections with a person after death are oftentimes or occasionally present. It has been discussed that these ties are inherent and remain over time as homages or rituals that exert an influence on the grieving person’s life. It is a broad phenomenon that would be linked to grief adaptation or non-adaptation. The expressions were dynamic, letting themselves show in the physical longing for the deceased person or in the mental evocation of their presence at the time of the loss and over time^(^ 19 ^)^.

It has been deduced that internalized CBs are an inner representation of the deceased person with which the bond is maintained through thoughts, by feeling their presence or by believing that it exerts a positive influence; in turn, externalized CBs are distinguished in activities at the beginning of the grieving process, such as illusions or hallucinations. It has been reasoned that internalized CBs predict positive results, whereas externalized ones predict negative results in solving sorrow; however, the role of CBs depends on the capacity to accept death and on the reconstruction of meanings. In this specific case, we would not ignore that what the participants revealed is endowed with infinity of existential symbolisms that would hinder a single interpretation^(^ 20 ^)^.

The differences evidenced in terms of internalized and externalized CBs by degree of kinship with the deceased person are consistent with what was observed in other findings. In this sense, the value attributed to kinship by the grieving person would deliberatively influence the types and degrees of CBs. According to internalized CBs, the deceased person would become a trust model; in addition, in externalized CBs, they would remain bodily present in the world. Both cases increase the grief symptoms but it has been inferred that the quality of the relationship might change the intensity of CBs when the grieving person find consolation to their sorrow. It can be supposed that, in this collective of men, the representation of who the deceased person was would outdo other situations around their death^(^ 21 ^)^.

Referring to the time elapsed since the event, it is agreed with reports where no difference is established, which might allude to the participants’ personality traits and their environment. Nevertheless, it might be explained that the time elapsed might be a key element to understand CBs, as it is usual for grieving people to experience changing frequencies of thoughts and feelings marked by longing. Actually, it would be thought as normal and healthy; therefore, it might be supposed that sorrow would extend over time on some occasions, and it would be reasonable to state that its duration would not have to be defined^(^ 22 ^)^.

Divergences were found with the cause of death; other research studies have reported significant differences for internalized and externalized CBs. Actually, depending on the cause of death, it has been predicted what type of CBs would be experienced; for example, when the death was unexpected or violent, it has been linked to externalized CBs, making lesser use of internalized CBs when compared to natural deaths. Thus, the death scenario disorganizes the normal life order, exerting an influence on the grief manifestations. The results strengthen the need to expand the study of the differences about internalized and externalized CBs among the mediators of mourning^(^ 22 ^-^ 23 ^)^.

In opposition to the aforementioned in relation to the associations between CBs and mediators of mourning, various research studies have reported significant correlations of CBs with cause of death and time elapsed since the loss, which would support grief adaptation or non-adaptation. It has been presented that, when the cause of death generates distrust or insecurity, external bonds that cause difficulties would be expressed. Likewise, persistence of some internal and external bonds over time would be a synonym of non-adaptation^(^ 24 ^)^.

It has been proposed that, when a grieving person cannot manage to differentiate ideas in their mind from the external reality, they would show distress fostering non-adaptation; in opposition, grieving people who manage to interpret and understand their mind’s symbolisms would reach adaptation. It draws the attention that, in this group of men, these mediators of mourning would exert no influence on elaboration of the grief process, which would be supported on the idea that CBs are so complex a phenomenon that behave in a delayed way during the grieving process^(^ 24 ^-^ 25 ^)^.

However, there would be similarity in what has been disclosed about the relationship between CBs and degree of kinship with the deceased person in other studies, where no significant relationships were established. In conclusion, death is a stressful event that is accompanied by physiological and psychological reactions determined by elementary particularities such as before-and-after relationships. Although it has been evidenced that the quality of the bond with the deceased person is a protective factor of the grieving process and that closeness of such relationship along with the attachment forms would be associated with CBs, men might perceive, process and communicate their conception of the experience in different ways. The results might guide the duty of integrating the cultural differences to understand this population group in greater depth^(^ 25 ^-^ 26 ^)^.

To conclude, it might be argued that the findings would reflect the complexity of the grief process as CBs in men. For being an extremely intimate phenomenon, each population will have its own particular characteristics defining it and differentiating it from others. It might be thought that generalization is not in line with the actual needs; on the contrary, specificity favors depth, closeness and sensitivity towards grieving men’s sorrow.

The following stand out as important strengths: conveying the analysis of the grieving process as CBs (internalized and externalized) in men for being one of the most research studies in this area that would contribute new knowledge to understand the topic; in addition, the fact of using ECoVin as one of the first elements validated and adapted for Spanish to assess CBs, as well as the contribution of Nursing researchers who are experts in the topic.

However, the study is not exempt from limitations, which should be reported. Its cross-sectional design fails to establish causality and is not suitable for the evolutionary nature of grief, the recruitment plan might be vulnerable to self-selection biases, and the self-applied configuration of the data collection instrument might limit precision of the measurements. As the data collected are focused on grief in men, their confrontation might be limited because there are few existing studies with such specificity.

Some recommendations for research invite to conduct longitudinal studies that assess more mediators and variables to understand the role of CBs in the grief process among men, as well as studies that reveal men’s narratives about the meaning they attribute to the grief process as CBs.

According to the practice, it is recommended to devise specific promotion and prevention strategies based on scientific evidence for grieving men where markers such as masculinities, cultures and spiritualities are valued, as well as all those converging in grief coping and adaptation with the purpose of conferring meaning to CBs.

## Conclusion

CBs represent a complex open dialog of an intersubjective and interdependent nature that renders them unique and special for each grieving person immersed in a context with differentiated sociocultural characteristics. In this group of grieving men, the intensity with which internalized CBs are frequently expressed, and externalized ones on occasions, denotes a bond with the loved one marked by differences loaded with symbolisms according to who the deceased person was.
